# The relationship between umbilical cord blood IL-22 level and infantile eczema at 42 days

**DOI:** 10.3389/fped.2024.1376706

**Published:** 2024-03-28

**Authors:** Xujun Lu, Wenge Wang, Yang Wang, Chuo Huan, Yue Yang

**Affiliations:** ^1^ Department of Pediatrics, Air Force Medical University Air Force Medical Center, PLA, Beijing, China; ^2^ Department of Preventive Medicine, Wanshou Road Community Health Service Centre, Beijing, China

**Keywords:** interleukin-22, cord blood, infants, eczema, influence factors

## Abstract

**Background:**

The occurrence of eczema is related to helper T 22 (Th22) cytokine disorder, and Th22 mainly secretes interleukin-22 (IL-22). This study aims to investigate the predictive value of umbilical cord blood IL-22 levels on the onset of eczema in infants within 42 days.

**Study design:**

The study selected 157 full-term healthy neonates born between September 2020 and May 2021. Cord blood was collected immediately after birth to determine IL-22 levels, and the infants were followed up for 42 days to assess the incidence of eczema.

**Results:**

Among the 157 infants who completed the 42-day follow-up, 86 developed eczema and 71 did not. The level of IL-22 in the umbilical cord blood of the eczema group was lower than that of the non-eczema group (*p* < 0.05). Additionally, the incidence of eczema in children whose Family history of allergy was significantly higher than in the group without eczema (*p* < 0.05). Logistic regression analysis indicated that low cord blood IL-22 levels and a family history of allergies were independent risk factors for eczema (*p* < 0.05). The ROC curve of cord blood IL-22 levels and infant eczema showed that the cut-off value is 36.362 pg/ml, the area under the curve (AUC) is 0.613, the standard error is 0.045, the 95% CI is 0.526–0.701, the sensitivity is 63.4%, and the specificity is 57.0%. Therefore, there is a certain correlation between cord blood IL-22 levels and the incidence of infant eczema.

**Conclusions:**

Low IL-22 levels in umbilical cord blood may be linked to the development of infant eczema within 42 days, indicating a potential predictive value, although this value appears to be limited.

## 1 Introduction

Eczema is a chronic and recurring inflammatory skin disease. Clinically, atopic dermatitis and contact dermatitis have eczema-like symptoms (1, 2). In China, conditions such as milk tinea, infantile eczema, eczema, flexural dermatitis, and neurodermatitis are predominantly considered forms of AD. Thus, the distinction between eczema and AD is not well-defined, indicating that eczema could essentially be a manifestation of AD (3, 4). Atopic eczema [or atopic dermatitis (AD)] is the most common form of eczema in children, witnessing a yearly increase in incidence (5). The consequences of eczema surpass mere physical manifestations, impacting children's psychological health and placing financial strain on families. Research has demonstrated that persistent eczema can hinder the physical and mental development of children (6). Consequently, assessing early-life risks and devising prevention strategies continue to be the primary areas of focus for reducing the global burden of allergic diseases (7, 8).

The exact cause of eczema remains elusive. Initial studies posited that an imbalance between helper T1 (Th1) (e.g., interleukin-2, Interferon-γ (IFN-γ)) and Th2 (e.g., IL-4, IL-13) cells was a primary factor in the disease's development (9). Th2 cells are pivotal in the pathogenesis of eczema (10). For example, Dupilumab effectively treats eczema by targeting IL-4 and IL-13 secretion from Th2 cells (11). However, as research has evolved, this Th1/Th2 cell imbalance theory is increasingly seen as insufficient for a comprehensive understanding of eczema's pathogenesis (12). More recent studies have identified an independent T-helper cell subgroup, Th22, thereby opening a new avenue for researching the disease's origins (13). IL-22, part of the IL-10 family, is primarily secreted by Th22 cells and plays a role in skin inflammation, host defense, and tissue homeostasis. It is expressed in various tissues, including the skin, intestines, lungs, liver, kidneys, and pancreas (14). Earlier research posited that IL-22 was primarily secreted by Th17 cells and functioned in conjunction with IL-17 (15). Existing literature indicates that IL-22 may contribute to skin inflammation in eczema patients (16). More recent studies, however, indicate that IL-22 is predominantly produced by Th22 cells. IL-22 exacerbates eczema through binding to IL-22R and activating a range of signaling pathways, including extracellular regulated protein kinases (ERK), c-Jun N-terminal kinase (JNK), p38 mitogen activated protein kinases (p38MAPK), nuclear transcription factor-κB (NF-κB), and activator protein-1 (AP-1) (17–19).

Umbilical cord blood, an early and accessible biological fluid, may offer valuable insights into potential biomarkers for infant eczema. The 42-day mark, coinciding with infants’ first medical follow-up, presents an opportune moment for evaluating eczema incidence and aligns with diagnostic criteria. Yet, no studies have explored the correlation between IL-22 levels in umbilical cord blood and the onset of eczema in 42-day-old infants. This approach seeks to inform early prevention and treatment strategies, potentially reducing the severity or preventing the onset of eczema.

## 2 Materials and methods

### 2.1 Research subjects

Between September 2020 and June 2021, 157 full-term healthy newborns were born in the air force medical center of PLA.

Inclusion criteria:

1. Full-term newborns delivered in the obstetrics and gynecology department.
2. Normal Apgar score at birth.

Exclusion criteria:

1. Congenital malformations.
2. There was intrauterine distress before birth.
3. There are neonatal diseases such as hyperbilirubinemia and neonatal hemorrhage after birth.
4. Abnormal screening results of neonatal diseases such as congenital adrenocortical hyperplasia, congenital hypothyroidism, phenylketonuria and glucose-6-phosphate dehydrogenase deficiency.

This study was approved by the center's ethics committee, and all parents of the newborns provided informed consent. Ethical number [2023-28-PJ01].

### 2.2 Methods

#### 2.2.1 Procedures for Il-22 collection in neonatal umbilical blood

Immediately post-delivery, 5 ml of umbilical cord blood is collected from the placental end using a 10 ml syringe and promptly transferred to an anticoagulant collection vessel. The sample is then centrifuged at 3,000 rpm for 10 min. Subsequently, 1.5 ml of the upper plasma layer is drawn using a pipette and transferred into an EP tube. These samples are stored in a designated refrigerator at −80°C until all samples are collected to prevent the effects of repeated freezing and thawing on test outcomes.

#### 2.2.2 Measurement of Il-22 levels in neonatal umbilical blood

Cord blood was collected into EP tubes and processed as per the Enzyme-linked Immunosorbent Assay (ELISA) kit's instructions to determine IL-22 levels. IL-22 levels were specifically measured using a kit (batch number 330876-001) obtained from Semel Technology Co., Ltd., USA. Reagents and samples, initially refrigerated, were brought to room temperature before processing. Following the kit's protocol, the optical density (OD) of each sample was measured at a 450 nm wavelength using an enzyme labeling instrument. Subsequently, the IL-22 mass concentration was calculated using specific software.

#### 2.2.3 Collection of basic information

Data collection encompassed newborn gender, birth weight, mode of delivery, gestational age, and feeding method. Additionally, information was obtained on family history of allergies (asking if first-degree relatives have eczema, allergic rhinitis, or allergic asthma), maternal health during pregnancy (such as diabetes, hyperthyroidism). The study inquired about maternal dietary habits, including the consumption of fish (≥ twice a week), meat (≥500 g), and nuts during pregnancy.

### 2.3 Study design and follow-up survey

This research is a prospective study.

At 42 days post-birth, outpatient physical examinations were conducted to observe and record the presence of eczema in the infant.

Early diagnostic guidelines for infant eczema are as follows:

The eruption typically manifests within two weeks post-birth, accompanied by symptoms such as itching, irritability, or sleep disturbances correlating with the rash. A diagnosis of eczema can be confirmed if, in addition to these, the infant displays one or both of the ensuing criteria: eczema-like skin lesions located on the cheeks, scalp, or extremities, and/or eczema-like skin lesions on any other body part, frequently associated with xeroderma. Exclusion of other potential conditions, such as contact dermatitis, psoriasis, scabies, genetic or metabolic diseases, and lymphoma, is essential before confirming a diagnosis.

Eczema severity standard: Eczema Area and Severity Index (EASI) is used to evaluate the severity of eczema. Mild eczema: 0.1–7 points; moderate eczema: >7–21 points; severe eczema: >21–50 points; very severe eczema: >50 points.

### 2.4 Data processing and analysis

Data analysis was conducted using SPSS version 24.0. Measurement data following a normal distribution are presented as mean ± standard deviation (S ± D). These data sets were compared using the *T*-test. For non-normally distributed quantitative data, the quartile interval M[P50(P25, P75)] was employed, and the Mann-Whitney *U* test was utilized for comparisons. Categorical data are expressed as frequencies (percentages) [*n* (%)], and comparisons were made using a chi-square (*χ*^2^) test. Logistic regression analysis was applied to statistically significant factors, and a Receiver Operating Characteristic curve (ROC curve) was used to evaluate the predictive value of IL-22 levels for 42-day-old infant eczema. A *p*-value less than 0.05 was considered statistically significant.

## 3 Results

### 3.1 Comparison of standard influencing factors in newborns between groups

Following a 42-day follow-up, infants were classified as either belonging to the eczema or the eczema-free group, based on the manifestation of the condition. Analysis indicated a significant difference in the family history of allergies between the two groups (*p* < 0.05). However, no significant differences were observed in gender, birth weight, gestational age, delivery method, maternal health during pregnancy, consumption of meat, nuts, fish during pregnancy, or feeding methods (*p* > 0.05). For further details, see Table 1.

**Table 1:** Comparison of general influencing factors between two groups of infants.

	Eczema group (*n* = 86)	Non-eczema group (*n* = 71)	*X^2^/t*	*P*
Sex of newborn			0.461	0.497
Male	45 (52)	41 (58)		
Female	41 (48)	30 (42)		
Baby weight (g)	3,342.442 ± 393.963	3,257.042 ± 327.032	−1.458	0.147
Mode of delivery			0.966	0.326
Natural labor	62 (72)	46 (65)		
Cesarean section	24 (28)	25 (35)		
Gestational age (weeks)			3.714	0.156
<39	14 (16)	20 (28)		
39–40	51 (59)	39 (55)		
>40	21 (25)	12 (17)		
Maternal health status during pregnancy			0.616	0.432
Yes	37 (43)	36 (51)		
None	49 (57)	35 (59)		
Family history of allergy			4.526	0.033
Yes	51 (59)	30 (42)		
None	35 (41)	41 (58)		
Feeding pattern			3.329	0.068
Breast-feeding	42 (49)	45 (63)		
Mixed feeding	44 (51)	26 (37)		
Consumption of fish during pregnancy (≥twice a week)			0.006	0.936
Yes	49 (57)	40 (56)		
None	37 (43)	31 (44)		
Consumption of more meat during pregnancy (≥500 g)			1.155	0.283
Yes	58 (67)	42 (59)		
None	28 (33)	29 (41)		
Consumption of Nuts during pregnancy			0.944	0.331
Yes	55 (64)	40 (56)		
None	31 (36)	31 (44)		

### 3.2 Relationship between Il-22 Levels in umbilical cord blood and 42-day-old infant eczema

The statistical analysis indicated that the level of IL-22 in the umbilical cord blood of the eczema group was lower than that in the eczema-free group. This confirms that a low level of IL-22 in umbilical cord blood is associated with the onset of eczema (*p* <0.05), as depicted in Table 2.

**Table 2:** Comparison of IL-22 levels in umbilical cord blood.

Group	Number	IL-22 levels in umbilical cord blood (pg/ml)
Eczema group	86	31.767 (17.853, 442.330)
Non-eczema group	71	39.320 (25.415, 46.664)
Z value		−2.445
*P* value		0.015

### 3.3 Comparison of Il-22 Levels in children with eczema of different severity

The study revealed that infants at 42 days old displayed mild to moderate eczema. Statistical analysis showed no significant difference in IL-22 levels between the mild and moderate eczema groups (*p* > 0.05), as detailed in Table 3.

**Table 3:** Comparison of cord blood IL-22 levels between mild and moderate eczema.

Group	Number	IL-22 levels in umbilical cord blood (pg/ml)
Mild eczema group	73	30.439 (17.853, 41.503)
Moderate eczema group	13	40.906 (24.146, 45.668)
Z value		−1.429
*P* value		0.153

### 3.4 Logistic regression analysis of risk factors related to 42-day-old infant eczema

Logistic regression analysis confirmed that family history of allergy and low levels of IL-22 in umbilical cord blood were independent risk factors for infant eczema at 42 days old (*p* < 0.05). See Table 4 for details.

**Table 4:** Logistic regression analysis of influencing factors of infant eczema.

Independent variable	β Value	*S.E.*	*Wald/χ^2^*	*OR*	*95% CI*	*P*
IL-22 Levels in Umbilical Cord Blood	-0.032	0.012	7.448	0.968	0.946–0.991	0.006
Family history of allergy	0.801	0.338	5.614	2.228	1.148–4.321	0.018

### 3.5 Correlation between cord blood Il-22 Levels and 42-day-old infant eczema

**Figure 1:**
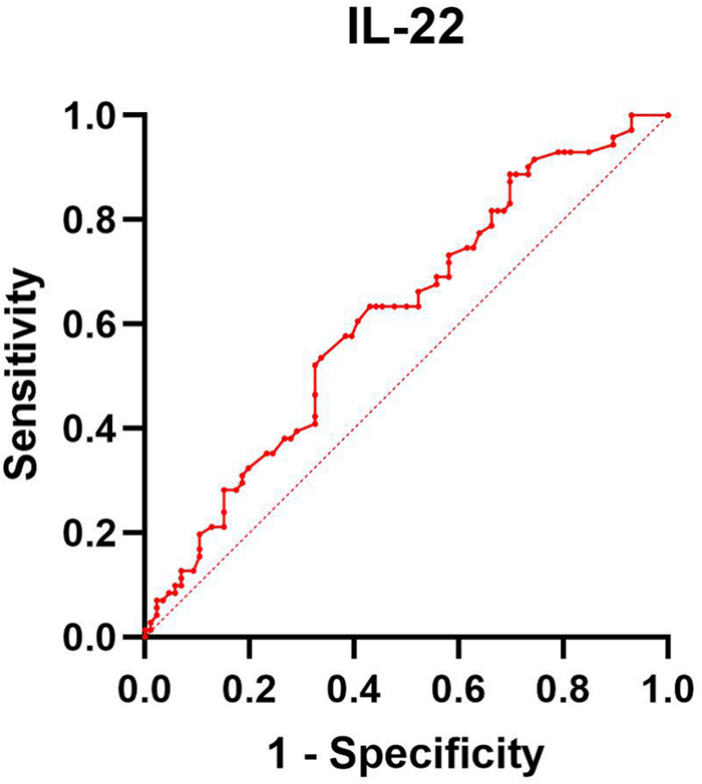
ROC curve of correlation between cord blood IL-22 levels and 42-day infant eczema.

ROC analysis for IL-22 was conducted, and the results are presented in Table 5 and Figure 1. This analysis indicates that IL-22 levels are a significant factor in the occurrence of infant eczema at 42 days old (*p* < 0.05).

**Table 5:** ROC analysis results of cord blood IL-22 levels.

Index	IL-22 levels in umbilical cord blood
Cut value	36.362 (pg/ml)
AUC	0.613
Standard error	0.045
95% CI	0.526–0.701
Sensitivity degree (%)	63.4
Specificity degree (%)	57.0

## 4 Discussion

Eczema, an immune-mediated skin disorder, affects 15%–30% of children worldwide, with its prevalence increasing (20, 21). Of the 157 subjects included in this study, 86 developed eczema within 42 days, while 71 did not, yielding an incidence rate of 54.8%. Although the underlying causes of eczema can only be speculated to involve environmental, genetic, and skin barrier factors (22), this study has established a significant link between a family history of allergies and the early onset of infant eczema (*p* < 0.05). Logistic regression analysis confirmed that a family history of allergies significantly increases the risk of developing early infant eczema, in agreement with existing literature. Research indicates that when both parents have allergic diseases, their children's risk of experiencing allergic diseases is 19.03 times greater than in families without a genetic history of allergies. When only the mother has allergic diseases, the risk for the children rises to 12.55 times (23). An Asian study demonstrated that children with a family history of allergies are at a higher risk of experiencing persistent wheezing, early allergic symptoms, and eczema (24).

IL-22, a member of the IL-10 family, is primarily produced by innate lymphoid cells (ILCs) and CD4+ T cells. The levels of IL-22 in the skin of children with eczema are considerably higher than in adults and correlate significantly with disease activity (25). Recent advancements have shown that inhibiting IL-22 expression can effectively treat eczema (26), and IL-22 has emerged as a crucial factor in eczema evaluation and treatment (27). RyomaKishi (28) et al. discovered a correlation between IL-22 levels and the severity of epidermal thickening and itching in eczema patients.

Consequently, we explored the potential role of IL-22 in eczema's etiology, hypothesizing that its levels in neonatal cord blood could serve as an early marker of immune function. Our findings revealed significantly lower IL-22 levels in the cord blood of infants with eczema compared to those without the condition. This suggests that reduced IL-22 levels could be a predictive factor for the early onset of eczema. However, this finding conflicts with prior studies that report elevated IL-22 levels in eczema patients. This discrepancy might arise from differences in cytokine production at different stages of immune development. Th17 cytokines dominate IL-22 production in early immune processes, and their levels in the cord blood of infants in the eczema group were lower than in those without eczema (8). Furthermore, our findings indicate that IL-22 levels do not correlate with the severity of early infant eczema, likely because the cases studied were predominantly mild to moderate. Logistic regression analysis and ROC curve results suggest that low umbilical cord blood IL-22 levels may play a role in the development of infant eczema within 42 days, indicating a limited predictive value. This limited correlation may be due to the predictive nature of the indicator, as eczema had not manifested at the time of measurement. Despite its modest predictive efficiency, this research is at the forefront of exploring early indicators for eczema, highlighting its innovative yet preliminary nature.

Nevertheless, the study has limitations. The sample size is small, and the IL-22 levels were not reassessed in infants at 42 days. The short follow-up period and inconsistent parental compliance also limit the data's accuracy. Moreover, selection bias was introduced as most participants were children of Beijing Army members. Future studies should increase the sample size, measure additional cytokines in cord blood, extend the follow-up period, and examine venous blood IL-22 levels at different ages to elucidate its role in eczema more comprehensively.

## 5 Conclusions

In summary, both low levels of IL-22 and Family history of allergy serve as risk factors for eczema in infants at 42 days old. Additionally, reduced levels of IL-22 in umbilical cord blood moderately predict the occurrence of 42-day infant eczema. However, several limitations should be acknowledged. The study had a small sample size and did not re-examine IL-22 levels in the venous blood of infants when they reached 42 days. The follow-up duration was brief, and non-compliance among some parents led to data inaccuracies. Moreover, a selection bias existed, as most participants were children of Beijing Army personnel. Future research should enlarge the sample size, measure additional cord blood cytokines, and extend the follow-up to include observations at 3, 6, and 12 months. Researchers should also collect venous blood at these time points to further investigate the role of IL-22 in eczema development.

## Data Availability

The original contributions presented in the study are included in the article/**Supplementary Material**, further inquiries can be directed to the corresponding authors.
